# Heterogeneous Motives in the Trust Game: A Tale of Two Roles

**DOI:** 10.3389/fpsyg.2016.00728

**Published:** 2016-05-18

**Authors:** Antonio M. Espín, Filippos Exadaktylos, Levent Neyse

**Affiliations:** ^1^Department of Economics and International Development, Middlesex University Business SchoolLondon, UK; ^2^Granada Lab of Experimental and Behavioral Economics, Departamento de Teoría e Historia Económica, Universidad de GranadaGranada, Spain; ^3^School of Agriculture Policy and Development, University of ReadingReading, UK; ^4^Bilgi Economics Lab of Istanbul, Murat Sertel Center for Advanced Economic Studies, Istanbul Bilgi UniversityIstanbul, Turkey; ^5^Kiel Institute for the World EconomyKiel, Germany

**Keywords:** Trust Game, dictator game, ultimatum game, social preferences, self-interest

## Abstract

Trustful and trustworthy behaviors have important externalities for the society. But what exactly drives people to behave in a trustful and trustworthy manner? Building on research suggesting that individuals’ social preferences might be a common factor informing both behaviors, we study the impact of a set of different motives on individuals’ choices in a dual-role Trust Game (TG). We employ data from a large-scale representative experiment (*N* = 774), where all subjects played both roles of a binary TG with real monetary incentives. Subjects’ social motives were inferred using their decisions in a Dictator Game and a dual-role Ultimatum Game. Next to self-interest and strategic motives we consider preferences for altruism, spitefulness, egalitarianism, and efficiency. We demonstrate that there exists considerable heterogeneity in motives in the TG. Most importantly, among individuals who choose to trust as trustors, social motives can differ dramatically as there is a non-negligible proportion of them who seem to act out of (strategic) self-interest whereas others are driven more by efficiency considerations. Subjects’ elicited trustworthiness, however, can be used to infer such motivations: while the former are not trustworthy as trustees, the latter are. We discuss that research on trust can benefit from adding the second player’s choice in TG designs.

## Introduction

Trustful and trustworthy individuals constitute an asset for their societies. Evidence documenting positive associations between general interpersonal trust levels or perceived trustworthiness and important country indicators such as economic prosperity, social equality, health, and happiness abounds (Kawachi et al., 1997; Knack and Keefer, 1997; Guiso et al., 2006, 2008; Oishi et al., 2011). But what exactly motivates individuals to act in a trustful and a trustworthy manner?

For many years and across disciplines, the consensus has been that trust is a form of encapsulated self-interest; an expression of a calculated risky decision based on the individual’s expectations regarding others’ trustworthiness. Similarly, trustworthiness has been considered a form of reciprocity with the purpose of maintaining mutually beneficial relationships (Rotter, 1980; Barber, 1983; Gambetta, 1988; Hardin, 2002).

The development of experimental games, and in particular of the Trust Game (TG; Berg et al., 1995), allowed a more systematic study of the underlying motives of trusting and trustworthy behavior. In the TG, a first player (the trustor) has the option to send an amount of money to a second player (the trustee). Whatever amount the trustor decides to send is multiplied by a factor (normally between 2 and 4) before reaching the trustee. Then, the trustee can send part of this money back to the trustor. The decision of the trustor measures trust, while that of the trustee measures trustworthiness. This research agenda has produced some consistent findings and demonstarted that both trust and trustworthiness are multi-dimensional concepts, not always guided by self-regarding considerations.

First of all, it is clear that not all individuals who trust in the TG base their decision on positive expectations of others’ trustworthiness. ^1^In fact, it has been shown that more than half of the trusting trustors believe that they will not make any profit out of trusting (Ashraf et al., 2006; Fetchenhauer and Dunning, 2009; Dunning et al., 2014). Even after they are explicitly informed that trusting is not the profit-maximizing option, most individuals keep choosing to trust, which is at odds with their private monetary interest (Berg et al., 1995; Ortmann et al., 2000).

Equally robust is the finding that differences in risk preferences cannot fully account for differences in trusting behavior. In fact, the two are rarely correlated (Eckel and Wilson, 2004; Ashraf et al., 2006; Kanagaretnam et al., 2009; Ben-Ner and Halldorsson, 2010; Houser et al., 2010; Etang et al., 2011; Corgnet et al., 2015b). What is more, when presented with both a TG and a lottery designed to mimic the return and risk level of the TG, some individuals choose to trust in the TG but not the risky option in the lottery (Fetchenhauer and Dunning, 2009; Dunning et al., 2012). However, the opposite often happens as well, which has been traced back to “betrayal aversion” (Bohnet and Zeckhauser, 2004; Bohnet et al., 2008; Corcos et al., 2012; Fairley et al., 2014). The above suggests that the decision to trust is something more or at least something different than simply a risky investment.

Scholars have early on tested whether these deviations from the “risky-investment” model can be explained, at least partially, by the trustors’ *social preferences*. Indeed, individuals who are generous in a Triadic Dictator Game – a game identical with the TG, but with the option of the trustee removed – are also more trustful in the TG (Dufwenberg and Gneezy, 2000; Cox, 2004). Likewise, individuals with prosocial Social Value Orientations (SVO)^2^ are more trusting compared to proself individuals (Kanagaretnam et al., 2009; Derks et al., 2014). Based on these results, individuals’ unconditional kindness or prosociality has been suggested as a factor prompting them to act in a trustful manner. However, unconditional kindness is a rather ambiguous term through the lens of social preferences and in fact a wide range of motives can explain generous, kind behavior (Charness and Rabin, 2002; Corgnet et al., 2015a).

Interestingly, unconditional kindness has been suggested to motivate the decisions of many trustees as well. While studies generally confirm the “traditional” reciprocity account of trustworthiness – reporting a positive slope between the amount sent by trustors and the amount returned by trustees (Güth et al., 2000; Schotter and Sopher, 2006; Bellemare and Kröger, 2007; Bornhorst et al., 2010) – trustees’ trustworthy behavior is in fact also predicted by their own behavior in a standard Dictator Game (Forsythe et al., 1994). In other words, kind individuals – those who are generous in a Dictator Game – also appear to be trustworthy in the TG (Ashraf et al., 2006; Chaudhuri and Gangadharan, 2007; Kovacs and Willinger, 2013). Note here again that generous offers in the Dictator Game can be triggered by either altruistic or egalitarian motives (Fehr and Schmidt, 1999; Staffiero et al., 2013). Additionally, antisocial motives such as spitefulness can lead to *seemingly* selfish, zero offers (Brañas-Garza et al., 2014), that is, to a *lack* of kindness.

Last but not least, the most trusting individuals are usually also the most trustworthy ones (or vice versa). That is, when individuals play both roles in the TG, their two decisions are highly correlated (Glaeser et al., 2000; Chaudhuri and Gangadharan, 2007; Altmann et al., 2008; Kovacs and Willinger, 2013). This evidence has been taken to suggest that there is probably a common factor informing both decisions. Combining this finding with the above arguments, such common factor might partly be related to individuals’ distributional (or outcome-based) social preferences, that is, their preferences over mere payoff distributions. Indeed in real-life social relationships, individuals typically act as both trustors and trustees when interacting with each other. It is thus plausible that people treat both roles as the two sides of a single strategy: how to act in social exchange relations.

Summarizing so far, the literature provides clear evidence that trust cannot be understood by beliefs and risk preferences alone, and that social preferences play an important role in both trusting and trustworthy behavior in TG. The literature nevertheless has not explicitly studied the particular social preferences that may account for TG strategies across roles, often silently assuming unconditional kindess or prosociality in general.

Theoretical advances on social preferences, however, have identified and formally defined a large array of motives that are widespread across individuals (Fehr and Schmidt, 2006; Van Lange et al., 2013). This paper takes a closer look on the kind of social preferences that may lead to different strategies in the TG. We focus on those social preferences that may rationalize trusting and trustworthy behavior from an outcome-based viewpoint. In particular, we consider preferences for altruism, spitefulness, egalitarianism, and efficiency. To these, we add narrow self-interest (or “selfishness”) and strategic self-interest as possible behavioral drivers.

### Social Motives in the Trust Game

*Altruism* refers to a positive concern for others’ payoffs (Andreoni and Miller, 2002; Fehr and Schmidt, 2006). Altruism may lead individuals to both trust and be trustworthy in the TG because such choices increase the payoff of their counterpart. But it is also true that increasing the payoff of the trustee can come at a disproportionately high (expected) cost for the trustor if the former is expected to return a low amount of money. Beliefs can therefore be important for an altruist’s decision to trust. In fact, while there is evidence that suggests a positive effect of altruism on trustworthiness (Ashraf et al., 2006; Chaudhuri and Gangadharan, 2007; Kovacs and Willinger, 2013), previous results regarding its impact on trust are less consistent (Dufwenberg and Gneezy, 2000; Cox, 2004; Brülhart and Usunier, 2012).

*Spitefulness* (Kirchsteiger, 1994; Fehr and Schmidt, 2006), on the dark side of social preferences, refers to a negative concern for others’ payoffs. Spitefulness may drive individuals to be untrustful and untrustworthy in the TG, in a analogous way that altruism drives them in the opposite direction: not trusting and not being trustworthy will result in minimizing the counterpart’s payoff and thus in the highest relative standing for the decision maker (Fehr et al., 2008).

*Egalitarianism* or aversion to inequality (Fehr and Schmidt, 1999; Van Lange, 1999) may lead individuals to be trustworthy in the TG because such choice is usually associated with an egalitarian outcome (Ciriolo, 2007; Corgnet et al., 2015b). The impact of inequality aversion on trusting behavior is more ambiguous, however. On the one hand, such an impact would depend on the trustor’s expectations about the trustee’s trustworthiness. On the other hand, it also depends on the initial endowments of the players. If the two players start the game with identical amounts of money (as in Berg et al., 1995), trusting would lead to a more unequal outcome than not trusting for any realistic expectations of trustworthiness – except when the trustor believes that every trustee will return exactly half of the money generated in the exchange – so that an egalitarian trustor should not necessarily pass the money. If the trustor starts with more money than the trustee (as it happens in many variants of the original TG, such as in that developed by Ermisch and Gambetta, 2006), then the expected trustworthiness is crucial to guide the behavior of an egalitarian trustor. Indeed, previous results have failed to find a clear effect of inequality aversion on trusting behavior (e.g., Dufwenberg and Gneezy, 2000; Bohnet and Zeckhauser, 2004; Cox, 2004; Bohnet et al., 2008; Brülhart and Usunier, 2012; Corgnet et al., 2015b).

*Efficiency* concerns or a preference for maximizing the total surplus (Van Lange, 1999; Charness and Rabin, 2002), may prompt individuals to trust in the TG since the pie is getting bigger as the trustor chooses to trust (see Bolton and Ockenfels, 2010). In principle, assuming that utilities are approximately linear over the relevant range of payoffs, as it is the typical assumption when dealing with experimental data (e.g., Fehr and Schmidt, 2006), a preference for efficiency should not motivate trustees’ choices since they cannot affect the total surplus.

Finally, self-interest can be driving choices in both roles of the TG. Trustors who are *narrowly selfish* in the sense that they believe that the trustee is selfish as well (so that she will not return any money, as the traditional self-interest model predicts) should not trust. On the other hand, *strategic* considerations could lead self-interested trustors to trust provided that they expect trustees to return at least some minimum amount. In the case of trustees, both narrow and strategic self-interest will dictate to return nothing.

**FIGURE 1:**
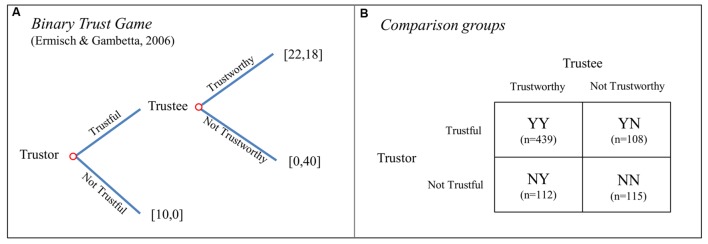
**Trust Game Strategies. (A)** Describes the binary Trust Game while **(B)** depicts the classification of the strategies (number of subjects in each group in parentheses).

We report data from a citywide survey-experiment where all participants in the sample (*N* = 774, after excluding incomplete observations) played a binary version of the Trust Game (**Figure 1A**), both as trustors and as trustees. Following the previous arguments regarding the dual-role nature of social relations, individuals were classified into one of four groups, each corresponding to a single strategy profile (see **Figure 1B**): (YY) Trustful & Trustworthy (56.72% of the sample), (YN) Trustful & Not Trustworthy (13.95%), (NY) Not Trustful & Trustworthy (14.47%), and (NN) Not Trustful & Not Trustworthy (14.86%). The participants also played a Dictator Game (DG) and an Ultimatum Game (UG; Güth et al., 1982). Based on these decisions, we were able to explore the social motives associated with each TG strategy. For our analysis, we start from the assumption that most people are consistent in their social preferences across games, which has been supported by recent empirical evidence (Yamagishi et al., 2013; Carlsson et al., 2014; Peysakhovich et al., 2014). However, we must note that individuals may switch strategies across games for instance as a result of moral licensing, a possibility that we cannot exclude (Monin and Miller, 2001; Dunning, 2007; Brañas-Garza et al., 2013; but see Gneezy et al., 2012, who find that licensing is less of an issue when social/moral decisions are “costly,” as it is the case in our design).

## Materials and Methods

### Ethics statement

All participants were informed about the content of the study prior to participating. Identical instructions were read aloud by the interviewers. Since literacy was not a requirement to participate (this was necessary to obtain a representative sample), we could not ask participants to read and sign the IC. Oral informed consent was obtained from all the participants included in this paper. Only those who accepted were allowed to participate. Anonymity was always preserved (in compliance with Spanish Law 15/1999 on Personal Data Protection) by randomly assigning a numerical code to identify the participants in the system. No association was ever made between their real names/addresses and the results. As is standard in socio-economic experiments, no ethical concerns are involved other than preserving the anonymity of the participants. No deception was used. This procedure (including the consent process) was checked and approved by the Vice-dean of Research of the School of Economics of the University of Granada; the institution hosting the experiments. At that time, there was no official IRB committee at the School of Economics.

### Design and Protocol

The survey-experiment took place in Granada (Spain) during the months of November and December 2010. A stratified sample of the city’s adult population (age ≥ 16) was recruited resulting in a total of 835 individuals. Participants completed the survey-experiment at their households upon agreement of participation in the presence of two monitors (there were a total of 108 pairs of interviewers). The resulting sample was representative of the city’s population both geographically and in terms of key demographic characteristics (see Appendix Table A1 and the supplementary information in Exadaktylos et al. (2013) for further details). One out every 10 participants was randomly selected to get paid. Average earnings among the selected participants were €9.60.

In the first part, a rich set of demographic and socio-economic information was collected. In the second part, participants played three experimental games, summing up to a total of five experimental decisions: a Trust Game (both roles), a Dictator Game (both roles, but one is passive), and an Ultimatum Game (both roles). For each decision, participants were randomly matched with a different anonymous participant. To control for possible order or spillover effects, the order both between and within games was randomized across participants, resulting in 24 different orders (always setting aside the two decisions of the same game). A double-blind procedure was implemented by having respondents privately marking their decisions on a response card and subsequently placing it into an envelope. Matching and payment took place a few days later. All procedures were common knowledge. See Appendix for more details.

### Binary Trust Game

For the measurement of trust and trustworthy behavior, a binary version of the TG developed by Ermisch and Gambetta (2006) was employed (see **Figure 1A**). In this version of the game, a first player, the trustor, is endowed with €10 and has to decide between (1) keeping the whole amount for herself (i.e., being not trustful) and (2) transferring the whole amount to the second player, the trustee (i.e., being trustful). In the former case, the game ends and the trustee gets a zero payoff. In the latter case, the €10 is being quadrupled, and consequently the trustee receives €40. Then the trustee has to decide between (1) keeping the whole €40 for herself (i.e., being not trustworthy), leaving the trustor with a zero payoff and (2) giving back €22 to the trustor and keeping €18 for herself (i.e., being trustworthy). All participants made a decision for both roles; for the trustee’s decision they reported what they would choose in case the trustor decided to pass the €10.

This version of the game is designed in a way that facilitates its application outside the lab, embedded in surveys (for a detailed description, see (Ermisch and Gambetta, 2006) and for an application see Ermisch et al., 2009). Importantly, it allows identifying trust and trustworthiness behavior in a simple and rigorous way. In particular, this design is well suited for the purposes of the present study, since it makes possible to build a two-by-two categorization as shown in **Figure 1B**.

### Constructing the Behavioral Motive Profiles

The behavioral motive profiles were constructed using participants’ decisions on the DG and the UG. In particular, in both games participants had to split a €20 pie between themselves and another anonymous participant. They decided which part of the €20 (in €2 increments), if any, they wanted to transfer to the other participant. In the case of the UG, implementation was upon acceptance of the proposer’s offer by the randomly matched responder; in case of rejection neither participant earned anything. For the role of the responder in the UG the strategy method was employed (Mitzkewitz and Nagel, 1993), in which subjects had to state their willingness to accept or reject each possible proposal beforehand. Based on these decisions, we calculated the Minimum Acceptable Offer (MAO) as responder of each participant.

Descriptive statistics for each of the three DG/UG raw behavioral measures can be found in Appendix Table A1, and the full distribution of choices is displayed in Appendix Figure A1. It can be seen that the equal split (€10) was the modal response in the three roles considered, whereas mean values were 39% of the pie (€7.86) for DG offer, 47% (€9.31) for UG offer and 35% (€6.99) for UG MAO. These behavioral patterns are similar to those observed in other representative and field studies (e.g., Bellemare and Kröger, 2007; Henrich et al., 2010).

As is the case with the TG, different motives may underlie individuals’ choices in these two games as well (see Staffiero et al., 2013; Yamagishi et al., 2013, 2014; Brañas-Garza et al., 2014; Peysakhovich et al., 2014; Espín et al., 2015). Based on the potential multiplicity of motives, we built a number of variables aimed at proxying how concerned an individual is regarding each motive (see **Table 1**). In particular, using subjects’ behavior in the DG and the UG, we defined six dummy variables to be used in explaining TG choices. Note that these variables are not based on incontestable or even mutually exclusive definitions (although sometimes this is the case; see Section “Statistical Analysis”), nor are they intended to be used to infer aggregate levels of such motives in the population. Also, we do not claim that individuals are concerned with just one single motive since, within subjects, multiple motives might be at play at the same time. Rather, these proxies are to be understood as simple empirical tools that may nonetheless be very helpful in uncovering the motives that are *relatively* more likely to be driving one, compared to another specific choice in the TG.

**Table 1:** Behavioral motive profiles.

Motive	Description (out of a pie of €20)
*Altruism*	(DG offer & UG offer ≥ 10) & (UG MAO = 0)
*Spitefulness*	(DG offer = 0) & (UG MAO = 10)
*Egalitarianism*	(DG offer & UG offer = 10) & (UG MAO = 10)
*Efficiency*	(UG offer > UG MAO)
*Strategic self-interest*	(UG offer > DG offer) & (UG offer < 20)
*Narrow selfishness*	(DG offer = 0) & (UG MAO ≤ 2)

In the case of the DG, as mentioned earlier, either egalitarianism or altruism can trigger generous offers, whereas zero offers may be the result of either selfishness or spitefulness. Something similar happens to the UG responder: a high MAO can be the result of either egalitarianism or spitefulness (Brañas-Garza et al., 2014) while a low MAO could arise from either selfish or, especially when MAO is set to zero (Staffiero et al., 2013), altruistic considerations. In this vein, we consider that *altruism* motivates those individuals who offered 10 or more out of the €20 in both the DG and the UG *and* who set their UG MAO to zero. *Spitefulness* would lead individuals to offer zero in the DG and set their UG MAO to the equal split (i.e., €10), whereas *egalitarians* split the pie equally in both games and set their MAO to half of the pie (**Table 1**; see also Brañas-Garza et al., 2014).

For UG proposers, apart from the above motives that may underlie DG giving as well, generous offers can also emanate from efficiency concerns and from strategic self-interest, both motives grounded in the avoidance of rejection. Given that a rejection destroys the pie, an individual motivated by efficiency should leave at least some “margin for agreement” between what she offers and what she demands in order to avoid rejections (Espín et al., 2015). Thus, we will characterize as *efficiency* concerned those individuals whose offer in the UG exceeded their own MAO. On the other hand, a straightforward measure of strategic behavior can be obtained by comparing the same participant’s offers in the DG and the UG (Steinbeis et al., 2012; Exadaktylos et al., 2013): we will consider that an individual is motivated by *strategic self-interest* if her offer in the UG is strictly higher than her offer in the DG (but excluding those individuals who offered the whole pie in the UG as they cannot have increased their offer from the DG to the UG out of self-interest; see **Table 1**). Finally, (narrowly) *selfish* individuals would offer zero in the DG and set their UG MAO either to zero or to the lowest positive amount (in our case, €2; these are the choices predicted by the self-interest model, see Fehr and Schmidt, 1999; Staffiero et al., 2013).

In A1, we show the proportion of subjects that can be classified according to each of these definitions. It is interesting to see that less than 4% of them displayed pure *selfishness* across games (a similarly low proportion has been found for instance in Yamagishi et al., 2014), whereas almost one half left some margin for agreement in the UG in order to avoid reducing *efficiency* through rejection. A similar proportion of all subjects can be classified as being motivated by altruism and spitefulness (about 8%), and by egalitarian and strategic concerns (roughly 30%).

### Statistical Analysis

For the statistical analysis we employed Stata 13 software. We use a multinomial logistic regression model where the categorical dependent variable describes in which group each participant was classified according to his/her decisions in the two roles of TG (as shown in **Figure 1B**). This allows us to explore the factors that impact relatively more on one TG strategy compared to the others. Regression analysis also permits us to control for a set of demographics and possible confounds.

The “Results” Section is divided in two parts. We first analyze the TG strategies as a function of the raw game measures (DG offer, UG offer, and UG MAO). Although these relationships have been studied before (e.g., Ashraf et al., 2006; Chaudhuri and Gangadharan, 2007; Yamagishi et al., 2012), the analyses so far have studied either the trustor’s or trustee’s decisions separately. In the first subsection, we contribute to this literature by considering both roles simultaneously.

In the second subsection, we employ the social motives (**Table 1**) as explanatory variables. Note that not all motives are mutually exclusive. The social motives that are independent to each other by definition are altruistic, spiteful, egalitarian, and selfish, on the one hand, and egalitarian and strategic on the other. The inclusion of all variables in the regression at the same time serves to investigate the relative incidence of each motive, controlling for the impact of the others that are not mutually exclusive. Although we have used definitions based on previous literature, as mentioned, these can be contested as there could be other ways to define the motives. However, we consider that our methodology is appropriate. Take the example of strategic self-interest and efficiency definitions. It could be argued that selfishly strategic individuals may have UG offer > UG MAO, which is the condition we imposed to define efficiency considerations (see also Espín et al., 2015). Indeed, 55% of those participants classified as strategic according to our definition, also belong to the efficiency category. Yet, note that this emanates from their high UG offer rather than their low MAO, since the latter does not reflect strategic behavior of a self-interested individual (i.e., low MAO is a condition for narrow, not strategic self-interest). Thus, we consider that strategic self-interest is more accurately defined by the difference between UG offer and DG offer (Steinbeis et al., 2012; Exadaktylos et al., 2013). Therefore, since we include all motives together in the regressions, any significant effect of efficiency is not due to strategic self-interest because the possible overlap is accounted for.

## Results

### Game Decisions

**FIGURE 2:**
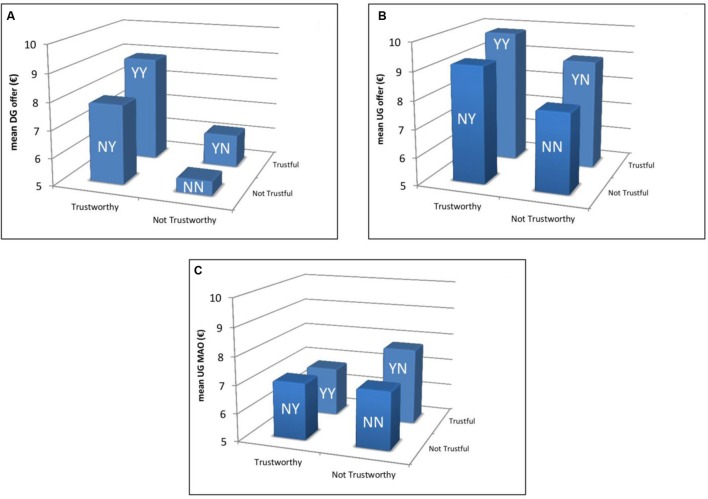
**Mean DG and UG behavior across TG groups. (A–C)** Refer to DG offer, UG offer, and UG MAO, respectively. As can be seen in **Figure 1B**, the number of observations in each group are 439 in YY, 108 in YN, 112 in NY, and 115 in NN.

**FIGURE 3:**
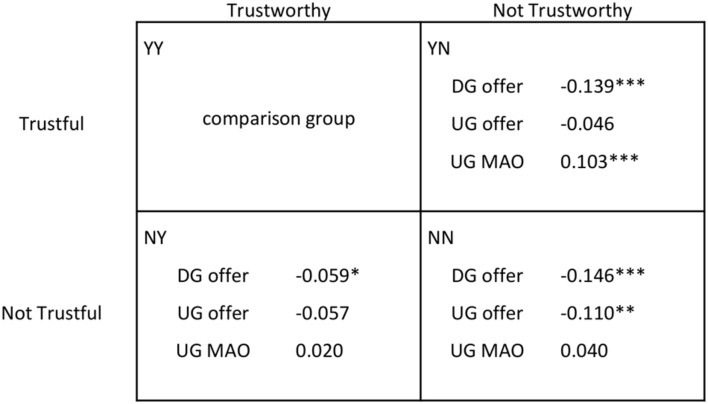
**TG outcomes as a function of DG and UG behavior.** Multinomial logit output. A positive (negative) coefficient means that individuals with a higher (lower) value of that specific variable (i.e., DG offer, UG offer, or UG MAO) are more likely to be included in that specific group (i.e., YN, NY, or NN) compared to group YY. Controls are: gender, age, household income, educational level, cognitive skills, risk preferences, and order effects. Complete regressions comparing all groups as well as regressions without controls can be found in Appendix Table A2. *^∗^p <* 0.10*, ^∗∗^p <* 0.05*, ^∗∗∗^p <* 0.01.

In **Figure 2**, we show for each of the four TG groups the mean values (in €) of the raw measures obtained from the DG and the UG, that is, DG offer (**Figure 2A**), UG offer (**Figure 2B**) and UG MAO (**Figure 2C**). In order to compare the relative incidence of each measure across TG groups, we performed a multinomial logit regression model (robust standard errors clustered on interviewers), the results of which are summarized in **Figure 3**. In the regression, we controlled for a number of basic variables and possible confounds including gender, age, educational level, household income, cognitive skills, and risk preferences (risk preferences were elicited using hypothetical monetary incentives; see Appendix for a description of each variable). In **Figure 3**, we set up group YY (Trustful & Trustworthy) as the comparison category. Complete regressions comparing all groups, with and without controls are presented in Appendix Table A2.

It can be seen from **Figure 2** that group YY captures those subjects with the highest mean DG (8.87 out 20) and UG offers (9.81 out of 20) whereas the lowest mean values of these two variables (5.51 and 7.84, respectively) are observed in group NN (Not Trustful & Not Trustworthy). In the case of UG MAO the picture changes considerably since it is groups YY and YN (Trustful & Not Trustworthy) that comprise the lowest (6.77 out of 10) and highest (7.76) mean values, respectively.

The results of the regression model confirm these observations (**Figure 3**). In particular, the differences between groups YY and NN in terms of DG and UG offers are significant (*p <* 0.01 and *p* = 0.02, respectively). This suggests a possible impact of altruistic and/or egalitarian considerations. The difference in UG MAO between groups YY and YN is also significant (*p* < 0.01). Some intermediate comparisons reach significance as well (see Appendix Table A2). The case of group YN seems particularly interesting for two reasons: on the one hand, individuals belonging to this group offer relatively little in the DG but have the highest MAO, which could be an indication of either spitefulness or lack of altruism; on the other hand, compared with group YY, their UG offer is not as low as their DG offer, which could be an indication of strategic self-interest. Lastly, the fact that the highest UG offer and the lowest MAO are observed in group YY suggests that efficiency concerns may be important for individuals belonging to this group. Our next analysis addresses these issues by studying the relative incidence of the above-defined motive profiles on each TG group.

### Social Motives

**FIGURE 4:**
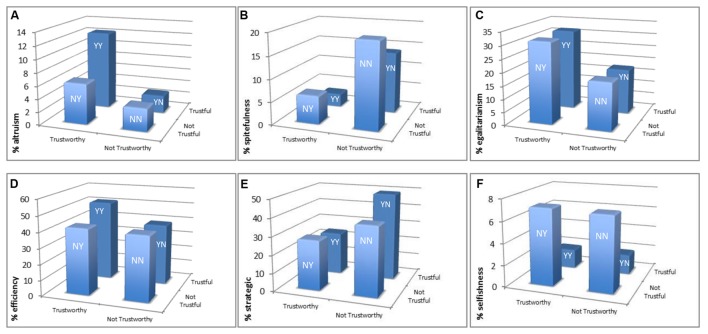
**Incidence of each motive profile across TG groups. (A–F)** Refer to altruism, spitefulness, egalitarianism, efficiency, strategic self-interest, and narrow selfishness, respectively. The percentages provided refer to the proportion of subjects within each group which can be classified according to each motive type. As can be seen in **Figure 1B**, the number of observations in each group are 439 in YY, 108 in YN, 112 in NY, and 115 in NN.

**FIGURE 5:**
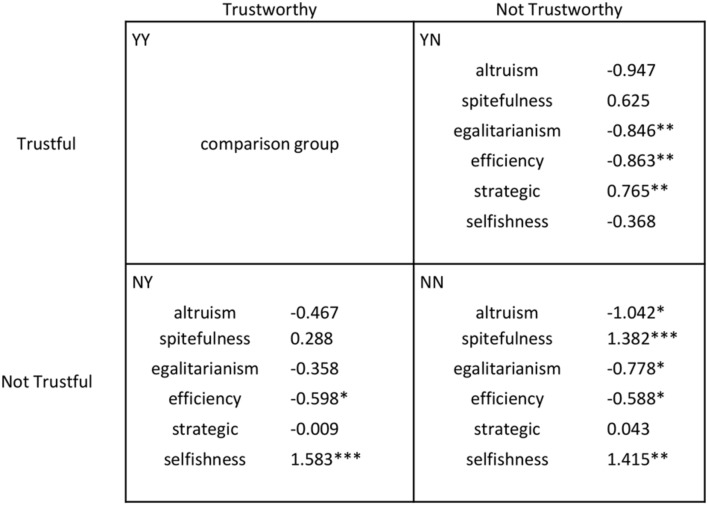
**TG outcomes as a function of motive profiles.** Multinomial logit output. A positive (negative) coefficient means that individuals with a higher (lower) value of that specific variable (i.e., the motive profile) are more likely to be included in that specific group (i.e., YN, NY, or NN) compared to group YY. Controls are: gender, age, household income, educational level, cognitive skills, risk preferences, and order effects. Complete regressions comparing all groups as well as regressions without controls can be found in Appendix Table A3. *^∗^p <* 0.10*, ^∗∗^p <* 0.05*, ^∗∗∗^p <* 0.01.

In **Figure 4**, we display the proportion of subjects that can be classified according to each of the six motive profiles obtained from DG/UG behavior, broken down by TG groups, as before. **Figures 4A–F** refer to altruism, spitefulness, egalitarianism, efficiency, strategic self-interest, and narrow selfishness, respectively. To statistically test the differences between TG groups, we again performed a multinomial logit regression model, which is summarized in **Figure 5**. Complete regressions comparing all groups, with and without controls are presented in Appendix Table A3.

With regards to *altruism*, **Figure 4A** shows that the highest percentage of subjects (12.30%) is concentrated in group YY, while the lowest incidence of altruism is observed in group YN (2.78%) closely followed by group NN (3.48%). However, the difference between groups YY and YN is only close to significance according to the regression results (*p* = 0.12; **Figure 5**). The only difference with respect to group YY that reaches (marginal) significance is that of group NN (*p* = 0.06), which is the strongest difference (i.e., it is associated with the highest absolute coefficient) in the regression output. Turning to *spitefulness* (**Figure 4B**), the highest incidence is observed in group NN (19.13%) while the lowest is observed in group YY (2.96%). This difference is indeed significant according to the regression (*p <* 0.01; **Figure 5**). These results seem to suggest that altruism is positively related with both trust (especially among trustworthy individuals) and trustworthiness (especially among trustful individuals) whereas spitefulness is negatively related with both, although more with trustworthiness.

When it comes to *egalitarianism*, **Figure 4C** shows that, while group YY has the highest incidence (31.66%), groups YY and NY comprise an almost identical proportion of subjects, as it happens with groups YN and NN, although the lowest incidence is observed in the former (17.59%). This symmetry indicates that egalitarian considerations only matter for trustworthy but not for trusting behavior. Indeed, only groups YN and NN are significantly different from group YY in terms of egalitarianism (*p* = 0.03 and *p* = 0.05, respectively; **Figure 5**). In the case of *efficiency* (**Figure 4D**), the highest difference is again observed between groups YY (51.25%) and YN (38.89%). Remarkably, groups NY and NN (both below 42%) comprise a very similar percentage of efficiency concerned individuals as group YN, and much lower than that in group YY. The regression results confirm these observations as the incidence of efficiency concerns is significantly lower in group YN (*p* = 0.03), and marginally significantly lower in groups NY and NN (both *p* = 0.08), compared to group YY (**Figure 5**). Thus, efficiency seems to be a concern for trust only among trustworthy individuals.

We now turn to self-interest. It can be seen from **Figure 4E** that *strategic* considerations are particularly prominent among individuals included in group YN (49.07%) while they are relatively more absent among those included in group YY (23.92%). This difference is significant according to our regression model (*p* = 0.02; **Figure 5**). Thus, strategic considerations are apparently relevant for trust only among those individuals who are not trustworthy as trustees (indeed, the difference between trustful and not trustful individuals among those who are trustworthy, i.e., groups YY and NY, is insignificant, *p* > 0.9). Lastly, with regards to the small percentage of subjects who can be classified as *narrowly selfish*, the incidence ranges from 1.82% in group YY to 7.14% in group NY (**Figure 4F**). Yet the proportions in groups YN and NN are nearly identical to those in groups YY and NY, respectively. According to the regression model, in fact, only groups NY and NN differ significantly from group YY (*p <* 0.01 and *p* = 0.01, respectively; **Figure 5**). This finding suggests that narrow self-interest only matters for trust but not for trustworthy behavior, although due to the low incidence of narrow selfishness we interpret this result with caution.

## Discussion

The paper presents a rich behavioral dataset from a large representative sample. The results reveal that our understanding of individuals’ behavior in the Trust Game can be largely benefited by having subjects play both roles in the game. One of the most striking results that emerged from the analysis is the considerable differences observed within trustful individuals: individuals who are both trustful and trustworthy (group YY) differ remarkably from those trustful individuals who are not trustworthy (group YN). Trust in the former group seems to be driven more by efficiency concerns and less by strategic considerations than the latter. What is more, the motivational gap between these two trypes of trustful individuals (YY vs. YN) seems to be more pronounced than that between group YY and group NY (not trustful but trustworthy individuals). In fact, regarding efficiency and especially strategic considerations – but not regarding altruism and spitefulness – the difference of YY with YN is even larger than with NN (not trustful and not trustworthy). Note also that among untrustful individuals the differences are much less pronounced (in fact groups NY and NN only differ significantly regarding one of the six motives under study: spitefulness impacts more on the latter; see Appendix Table A3). Interestingly, the only motive that is clearly related just to one single role is egalitarianism, which impacts on trustworthiness but not on trust.

This apparent distinction between trustful individuals of the YY and YN groups is particularly important given that the latter constitute a non-negligible 20% of all trustful individuals and might have serious implications for the way we interprete previous results. Consider for instance the case of the beneficial effects of trust at a macro level. The main theories regarding the causal mechanisms underlying these effects emphasize the “lubrication” of market interactions (Guiso et al., 2006, 2008, 2009) and the facilitation of group cooperation (Balliet and Van Lange, 2013a; Van Lange et al., 2013) and of norm-enforcement institutions (Balliet and Van Lange, 2013b). It would go a long way in either validating or discrediting each of these theories if we could know whether different trusting types are responsible for different processes. That is, are trusting individuals of one specific type more likely to be the catalysts of the cooperation-enhancing effects of trust? The answer is not obvious. It might be that these effects can be traced back solely to trustful individuals of the YY group because of their efficiency concerns. However, it might also be that the more strategic trustful individuals of the YN group are more responsive to the threat of sanctions and thus they increase cooperation to a larger extent when norm-enforcement institutions are at play. Analyzing these processes is an interesting endeavor for future research.

From an applied point of view, these results may also have interesting implications at a more micro level. Working teams could be organized in such a way so that those individuals who trust out of efficiency concerns are matched with those who are less trustworthy because the former will probably still cooperate with the latter in order to increase the team’s efficiency. Recall here that there is a considerable fraction of individuals who decide to trust even if they believe that their trust will not be reciprocated (Ashraf et al., 2006; Fetchenhauer and Dunning, 2009; Dunning et al., 2014). On the other hand, individuals who trust mainly due to strategic self-interest should be matched with trustworthy individuals since otherwise cooperation would likely collapse. Further research is needed on the interaction between trusting types and teamwork performance.

The present study also reveals that we might actually be able to distinguish between efficiency concerned and strategic trustful individuals by observing their behavior as trustees in the TG. Adding the trustee’s decision task to the research design might thus be a relatively inexpensive way of gaining potentially very valuable information. This is especially relevant since over the last years the TG is being used in many field studies (Barr, 2003; Schechter, 2007; Cárdenas and Carpenter, 2008; Johansson-Stenman et al., 2013) and is becoming increasingly embedded in large-scale surveys (Fehr et al., 2003; Bellemare and Kröger, 2007; Ermisch et al., 2009; Sapienza et al., 2013; Naef et al., 2014), substituting the the so-called trust-question.^3^

Lastly, these findings add to a growing literature challenging the traditional, often unidimensional interpretation of behavior in economic games, such as punishment in the Public Goods and Ultimatum Games and giving in the Dictator Game (Gächter and Herrmann, 2009; Espín et al., 2012; Yamagishi et al., 2012; Staffiero et al., 2013; Brañas-Garza et al., 2014).

A word of caution, however, is in order here. In this paper, we have analyzed the relative importance of different social motives for game play behavior in the TG. However, the aggregate incidence of these motives may change along with the change in expectations, for instance cross-culturally. That is, the expected behavior of the trustee may affect the importance of altruism, egalitarianism, and strategic self-interest for trusting behavior.

Apart from beliefs (and relatedly uncertainty), other motives might be at play at the same time. Indeed, some people might trust or be trustworthy just for the sake of trusting or being trustworthy. For example, Dunning et al. (2014) in a series of experiments provide evidence that trusting behavior follows the logic of social norms (that is, they are decisions one *should* make regardless of the material consequences; see, however, Bicchieri et al., 2011, suggesting that trustworthiness but not trusting behavior is a social norm). There is also plenty of evidence that immediate or anticipated emotions, such as shame, embarrassment, and excitement, may be driving trusting and trustworthy behavior in the TG as well (Loewenstein et al., 2001; Dunning et al., 2012; Schlösser et al., 2015). Additionally, a desire to signal (either to oneself or to others) that one is a good person, may also lead people to trust or to be trustworthy (Bodner and Prelec, 2003; Dunning, 2007; Barkan et al., 2015). Positive reciprocity, which falls outside the distributional social preferences analyzed here, is of course another important motive driving trustworthiness. Lastly, from a more methodological point of view, regarding our empirical strategy, one might argue that people are not using consistent strategies across games (as we essentially assume; Yamagishi et al., 2013) but are instead switching strategies across games following for instance moral licensing patterns (Monin and Miller, 2001).

## Author Contributions

All authors listed, have made substantial, direct and intellectual contribution to the work, and approved it for publication.

## Conflict of Interest Statement

The authors declare that the research was conducted in the absence of any commercial or financial relationships that could be construed as a potential conflict of interest.

## Footnotes

1 Note here that we use a parsimonious definition of trust as a behavior (Coleman, 1994). Such a behavioral definition leaves the reasons underlying trust open.

2 The SVO (Messick and McClintock, 1968; Van Lange et al., 1997) measures an individual’s preferences over monetary allocations between his/herself and another unknown individual. Measuring the SVO involves the individual making a number of (typically six or nine) distributional choices. Based on the these choices, the individual is classified as prosocial, individualist or competitor, with the latter two often collapsed into the proself category.

3 The question included in many surveys such as the General Social Survey and the World Values Survey reads as follows: *“Generally speaking, would you say that most people can be trusted or that you can’t be too careful in dealing with people?”*. Apart from issues of incentive incompatibility, and the vagueness in interpreting “trust”, there have been concerns regarding what the question actually measures (see Sapienza et al., 2013). Mirroring the traditional view of trust, the question indeed elicits the respondent’s expected trustworthiness about most people, rather than the respondent’s trust attitudes (in which case the wording would have been closer to “Do you trust most people?”). Therefore the cross-cultural evidence of the correlates of trust with economic and social indexes may in fact reflect something different than previously interpreted.
