# Supplementary material for: Heterogeneous Motives in the Trust Game: A Tale of Two Roles
Source: Front Psychol. 2016 May 18;7:728. doi: 10.3389/fpsyg.2016.00728 (PMC4870259; doi:10.3389/fpsyg.2016.00728)

Heterogeneous motives in the Trust Game: a tale of two roles

Antonio M. Espín, Filippos Exadaktylos, & Levent Neyse

Appendix

Procedures of the experimental games' decisions

In the second part of the survey experiment, all participants took a total of five experimental decisions. Special care was given in order to follow standard experimental practices. Apart from ensuring anonymity and privacy, all participants received some basic information regarding the decisions, without however revealing any specifics about each decision. In particular, participants were informed that they had to make five decisions, with the following protocol: (i) the five decisions involved real monetary payoffs coming from a national research project endowed with a specific budget for this purpose; (ii) the monetary outcome would depend only on the participant's decision or on both his/her own and another randomly matched participant's decision, whose identity would forever remain anonymous; (iii) one of every ten participants would be randomly selected to be paid, and the exact payoff would be determined by a randomly selected decision (role/game); (iv) matching and payment would be implemented within the next few days; (v) the procedures ensured double-blind anonymity by using a decision sheet, which they would place in the envelope provided and then seal. Thus, participants' decisions would remain forever blind in the eyes of the interviewers and the randomly matched participant. Once these general instructions had been given, the interviewer read the details for each experimental decision separately. After every instruction set, participants were asked to write down their decisions privately and proceed to the next task.

Control variables description

The control variables that are not self-explanatory are the following:

Educ: years of schooling. Categories: no studies (0), incomplete primary school (3), complete primary school (6), incomplete secondary school (8), complete secondary school (10), incomplete university diploma or technical degree (14), complete 3 university diploma or technical degree (15), incomplete bachelor or postgraduate degree (15), complete bachelor or postgraduate degree (17).

Income: average household monthly income in the last year (in €). Categories: 0, 500, 1000 , 1500, 2000, 2500, 3000, 3500, 4000, 4500.

*Cognit*ive skills: number of correct answers to the following five questions:

1. If the probability of being infected by an illness is 10%, how many persons of a group of 1000 would be infected by that kind of illness? (N if s/he cannot /do not want to answer).
2. If there are 5 persons that own the winning lottery ticket and the prize to be shared is two million Euros, how much money would each person receive?

3. Suppose that you have €100 in a savings account and the rate of interest that you earn from the savings is 2% per year. If you keep the money in the account for 5 years, how much money would you have at the end of these 5 years?: a. More than €102 b. €102 exactly c. Less than €102 d. S/he cannot/do not want to answer

4. Suppose that you have €100 in a savings account. The account accumulates a 10% rate of interest per year. How much money would you have in your account after two years?

5. The total cost of a bat and a ball is 1.10 Euros. The bat costs 1 Euro more than the ball. How many cents does the ball cost?

RISK PREFERENCES: dummies for "risk-loving" answers on each of the three following hypothetical questions (b, a and Y on questions Risk1, Risk2 and Risk3, respectively):

Risk 1. We flip a coin. Choose one of the following options: a. Take 1,000 Euros no matter if it is heads or tails. b. Take 2,000 Euros if it is heads and nothing if it is tails.

Risk 2. Choose one of the following options: a. Take a lottery ticket with 80% chance of winning 45 Euros and 20% chance of winning nothing b. Take 30 Euros

Risk 3. Would you accept the following deal? We flip a coin. If it is heads you win 1,500 Euros and if it is tails you lose 1,000 Euros: Yes (Y), No (N)

Table A1: Descriptive Statistics

**variables**	**mean**	**SD**	**min**	**max**
DG/UG raw measures				
DGoffer	7.858	4.324	0	20
UGoffer	9.307	9.307	0	20
UGmao	6.990	3.578	0	10
Motive profiles dummies				
Altruistic	0.088	0.283	0	1
Spiteful	0.074	0.261	0	1
Egalitarian	0.276	0.448	0	1
Efficiency	0.466	0.499	0	1
Strategic	0.301	0.459	0	1
Selfish	0.034	0.180	0	1
Controls				
Male	0.460	0.499	0	1
Age	37.694	17.065	16	89
Educ	12.010	4.518	0	17
Income	1912.791	1208.991	0	4500
Cognit	2.512	1.323	0	5
Risk1	0.138	0.345	0	1
Risk2	0.337	0.473	0	1
Risk3	0.090	0.287	0	1

Note: N = 774

Table A2. TG outcomes as a function of DG and UG behavior

	YN (vs. YY)		NY (vs. YY)		NN (vs. YY)		NY (vs. YN)		NN (vs. YN)		NN (vs. NY)	
	(1)	(2)	(3)	(4)	(5)	(6)	(7)	(8)	(9)	(10)	(11)	(12)
DGoffer	-0.139***	-0.147***	-0.059*	-0.049	-0.146***	-0.149***	0.080**	0.098**	-0.007	-0.002	-0.087***	-0.100***
	(0.030)	(0.027)	(0.031)	(0.032)	(0.030)	(0.031)	(0.038)	(0.038)	(0.038)	(0.033)	(0.033)	(0.034)
UGoffer	-0.046	-0.038	-0.057	-0.061	-0.110**	-0.134***	-0.011	-0.024	-0.064	-0.096	-0.053	-0.072
	(0.051)	(0.044)	(0.045)	(0.042)	(0.048)	(0.047)	(0.055)	(0.055)	(0.065)	(0.060)	(0.053)	(0.049)
UGmao	0.103***	0.088**	0.020	0.018	0.040	0.027	-0.084**	-0.070*	-0.064	-0.062	0.020	0.009
	(0.036)	(0.034)	(0.026)	(0.028)	(0.037)	(0.036)	(0.038)	(0.036)	(0.042)	(0.042)	(0.041)	(0.040)
Male	-0.459*	--	-0.005	--	0.169	--	0.454	--	0.628**	--	0.174	--
	(0.246)	--	(0.232)	--	(0.202)	--	(0.304)	--	(0.247)	--	(0.275)	--
Age	-0.023***	--	-0.003	--	-0.014**	--	0.020**	--	0.008	--	-0.011	--
	(0.008)	--	(0.007)	--	(0.005)	--	(0.009)	--	(0.010)	--	(0.008)	--
Educ	0.005	--	0.000	--	-0.002	--	-0.005	--	-0.008	--	-0.002	--
	(0.026)	--	(0.029)	--	(0.032)	--	(0.033)	--	(0.030)	--	(0.036)	--
Income	0.000	--	0.000	--	0.000	--	-0.000	--	-0.000	--	-0.000	--
	(0.000)	--	(0.000)	--	(0.000)	--	(0.000)	--	(0.000)	--	(0.000)	--
Cognit	-0.230**	--	0.016	--	-0.205*	--	0.246*	--	0.025	--	-0.221*	--
	(0.104)	--	(0.094)	--	(0.111)	--	(0.127)	--	(0.135)	--	(0.122)	--
Risk1	0.441	--	-0.096	--	0.632**	--	-0.538	--	0.191	--	0.729*	--
	(0.325)	--	(0.387)	--	(0.304)	--	(0.462)	--	(0.317)	--	(0.439)	--
Risk2	0.215	--	-0.404*	--	0.136	--	-0.619**	--	-0.079	--	0.540*	--
	(0.222)	--	(0.242)	--	(0.242)	--	(0.291)	--	(0.286)	--	(0.319)	--
Risk3	-0.198	--	-1.485***	--	-1.345***	--	-1.287*	--	-1.147*	--	0.140	--
	(0.361)	--	(0.582)	--	(0.535)	--	(0.661)	--	(0.614)	--	(0.775)	--
controls	yes	no	yes	no	yes	no	yes	no	yes	no	yes	no
constant	0.021	-0.577	0.014	-0.496	1.050	0.764	-0.006	0.081	1.029	1.341**	1.036	1.260**
	(1.128)	(.466)	(0.812)	(0.512)	(0.996)	(0.464)	(1.262)	(0.577)	(1.304)	(0.539)	(1.373)	(0.569)
ll	-779.233	-850.167										
pseu-R^2	0.1317	0.0526										
N	774	774										

Notes: Multinomial logit estimates. Robust standard errors clustered on interviewers (108 groups) are presented in parentheses. Regressions with controls also control for order effects. Regressions in columns (1), (3), and (5) are summarized in Figure 3 of the main text. * p < 0.10, ** p < 0.05, *** p < 0.01

Table A3. TG outcomes as a function of motive profiles

	YN (vs. YY)		NY (vs. YY)		NN (vs. YY)		NY (vs. YN)		NN (vs. YN)		NN (vs. NY)	
	(1)	(2)	(3)	(4)	(5)	(6)	(7)	(8)	(9)	(10)	(11)	(12)
Altruistic	-0.947	-1.141**	-0.467	-0.497	-1.042*	-1.104**	0.480	0.645	-0.095	0.037	-0.574	-0.608
	(0.608)	(0.574)	(0.441)	(0.450)	(0.554)	(0.517)	(0.679)	(0.699)	(0.732)	(0.734)	(0.707)	(0.664)
Spiteful	0.625	0.677	0.288	0.488	1.381***	1.505***	-0.337	-0.189	0.756	0.829*	1.093**	1.017*
	(0.522)	(0.419)	(0.498)	(0.485)	(0.472)	(0.436)	(0.576)	(0.510)	(0.551)	(0.437)	(0.536)	(0.519)
Egalitarian	-0.846**	-0.988***	-0.358	-0.368	-0.778*	-0.960**	0.488	0.619	0.068	0.028	-0.420	-0.592
	(0.387)	(0.357)	(0.374)	(0.362)	(0.403)	(0.388)	(0.422)	(0.424)	(0.502)	(0.485)	(0.453)	(0.453)
Efficiency	-0.862**	-0.787**	-0.598*	-0.531*	-0.588*	-0.567*	0.264	0.256	0.274	0.220	0.010	-0.035
	(0.399)	(0.350)	(0.340)	(0.318)	(0.338)	(0.302)	(0.419)	(0.379)	(0.443)	(0.405)	(0.358)	(0.324)
Strategic	0.766**	0.671**	-0.009	-0.111	0.043	-0.038	-0.775**	-0.782**	-0.723*	-0.709**	0.052	0.073
	(0.330)	(0.281)	(0.319)	(0.306)	(0.289)	(0.301)	(0.389)	(0.345)	(0.369)	(0.327)	(0.357)	(0.346)
Selfish	-0.368	-0.265	1.583***	1.533***	1.415**	1.426***	1.951***	1.798**	1.783***	1.691**	-0.168	-0.107
	(0.680)	(0.741)	(0.487)	(0.425)	(0.580)	(0.537)	(0.650)	(0.749)	(0.642)	(0.708)	(0.565)	(0.535)
Male	-0.526**	--	-0.016	--	0.170	--	0.510	--	0.696***	--	0.186	--
	(0.251)	--	(0.231)	--	(0.203)	--	(0.310)	--	(0.256)	--	(0.275)	--
Age	-0.021***	--	-0.003	--	-0.013**	--	0.019*	--	0.008	--	-0.011	--
	(0.008)	--	(0.007)	--	(-0.007)	--	(0.010)	--	(0.009)	--	(0.008)	--
Educ	0.010	--	-0.003	--	-0.011	--	-0.013	--	-0.022	--	-0.009	--
	(0.027)	--	(0.029)	--	(0.031)	--	0.034	--	(0.031)	--	(0.038)	--
Income	0.000	--	0.000	--	0.000	--	0.000	--	-0.000	--	-0.000	--
	(0.000)	--	(0.000)	--	(0.000)	--	(0.000)	--	(0.000)	--	(0.000)	--
Cognit	-0.214**	--	0.034	--	-0.175*	--	0.248**	--	0.039	--	-0.209*	--
	(0.102)	--	(0.095)	--	(0.106)	--	(0.125)	--	(0.126)	--	(0.119)	--
Risk1	0.517	--	-0.071	--	0.617**	--	-0.588	--	0.099	--	0.687	--
	(0.335)	--	(0.400)	--	(0.305)	--	(0.479)	--	(0.341)	--	(0.449)	--
Risk2	0.208	--	-0.437*	--	0.073	--	-0.645**	--	-0.135	--	0.510	--
	(0.229)	--	(0.243)	--	(0.244)	--	(0.298)	--	(0.297)	--	(0.318)	--
Risk3	-0.251	--	-1.498**	--	-1.342***	--	-1.247*	--	-1.091*	--	0.156	--
	(0.389)	--	(0.587)	--	(0.506)	--	(0.673)	--	(0.610)	--	(0.782)	--
controls	yes	no	yes	no	yes	no	Yes	No	yes	no	Yes	no
constant	-0.637	-1.002***	-0.660	-1.011***	-0.531	-0.930***	-0.023	-0.008	0.106	0.072	0.129	0.081
	(0.992)	(0.311)	(0.785)	(0.320)	(0.829)	(0.299)	(1.085)	(0.350)	(1.147)	(0.354)	(1.166)	(0.344)
ll	-776.270	-848.660										
pseu-R^2	0.1350	0.0543										
N	774	774										

Notes: Multinomial logit estimates. Robust standard errors clustered on interviewers (108 groups) are presented in parentheses. Regressions with controls also control for order effects. Regressions in columns (1), (3), and (5) are summarized in Figure 5 of the main text. * $p < 0.10$, ** $p < 0.05$, *** $p < 0.01$

Figure A1. Distribution of choices in the DG and UG

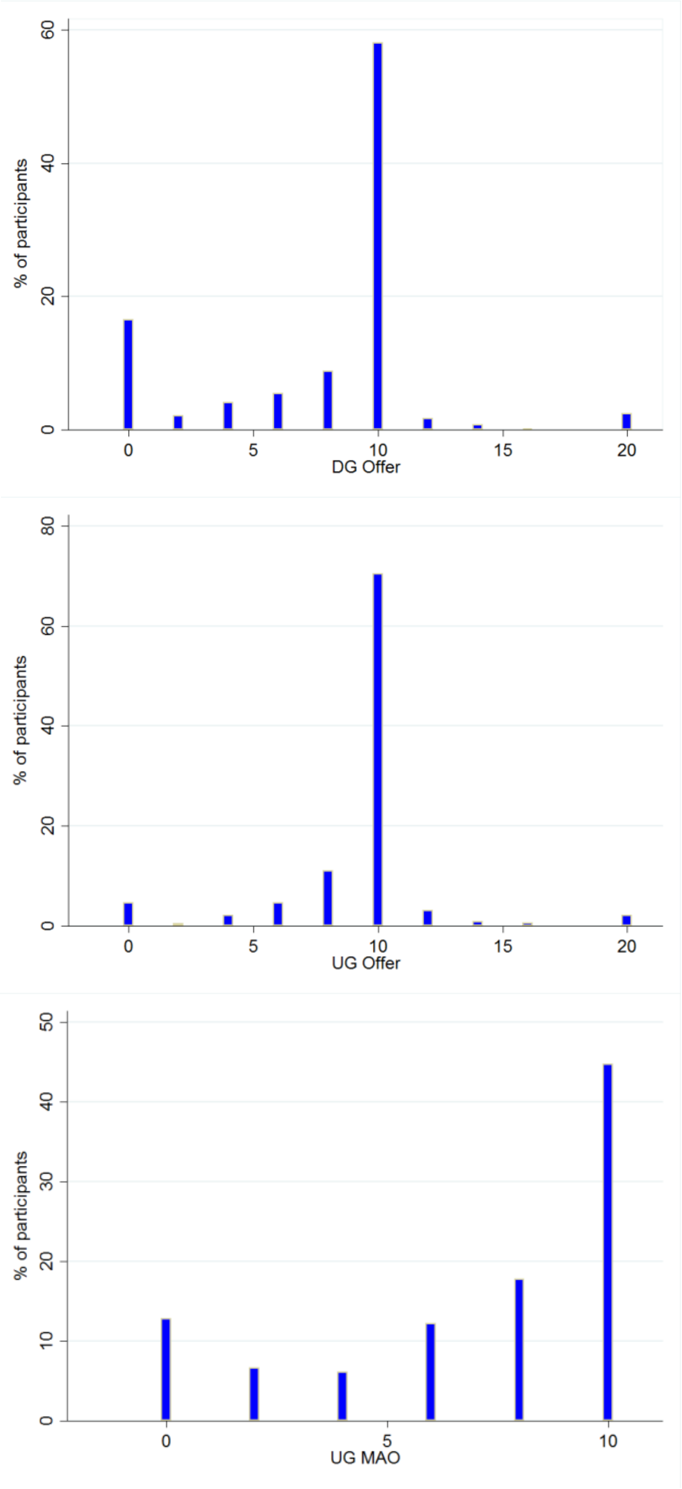

Supplement: Supplementary file 1 [file Data_Sheet_1.PDF]
